# Correspondence to: “Impact of pecto-intercostal fascial block on postoperative fatigue in elderly patients undergoing off-pump coronary artery bypass grafting: a randomized clinical trial”

**DOI:** 10.1097/JS9.0000000000002878

**Published:** 2025-07-15

**Authors:** Xiaolei Liu, BoHan Zhang

**Affiliations:** a Department of Orthopedics, The Fourth Affiliated Hospital of Nanjing Medical University, Nanjing, Jiangsu, China; b Department of Pain, The First Hospital of China Medical University, Shenyang, Liaoning, China

*Dear Editor,*

The recent research by Wang et al. on pecto-intercostal fascial block (PIFB) for postoperative fatigue syndrome (POFS) in elderly patients undergoing off-pump coronary artery bypass grafting (CABG) offers a novel focus on a critical postoperative outcome^[1]^. In accordance with transparent reporting standards, such as the TITAN Guidelines 2025, we confirm no artificial intelligence was used in this correspondence^[2]^. We propose alternative interpretations and future directions to strengthen these findings.

The temporary POFS reduction (days 1–5) coincides with ropivacaine’s 8–12-hour analgesia duration^[3]^. This suggests that continuous PIFB catheters would be more suitable for providing extended analgesia. Unaddressed neuroendocrine mechanisms, such as hypothalamic-pituitary-adrenal (HPA) axis dysregulation, and autonomic dysfunction might contribute to late-phase fatigue, but further studies are required to validate these hypotheses concerning PIFB’s effects^[4,5]^. PIFB generally reduces pain by lowering sympathetic activation and reducing inflammatory mediators such as IL-6 and TNF-α. This reduction helps mitigate early fatigue^[6]^. The reported decrease in hs-TnT and NT-proBNP could be due to diminished stress on the myocardium from pain control, but other factors such as the surgical technique or patient characteristics may also play a role. Functional data, such as echocardiography, are needed to confirm this interpretation^[7]^.

To broaden applicability, it is important not to exclude obese individuals with a BMI greater than 30 kg/m^2^ because ultrasound-guided PIFB is feasible in obesity. Inflammatory biomarkers are secondary outcomes that are underpowered and require multiplicity-adjusted or Bayesian analyses. Not providing justification for sample size concerning secondary outcomes, like inflammatory markers, increases the chances of Type II errors occurring, which means true differences that exist go undetected because there is not enough statistical power. We advocate for the use of continuous PIFB because it prolongs the analgesic effects^[3]^ and broader inclusion criteria for patients in subsequent trials. Evaluating sleep and mood could help pinpoint the drivers of late-phase fatigue in the postoperative context^[4]^. Wang et al.’s study also has typographical errors in Table 2 (e.g., “Preoperative 1d”), which should be corrected to not lose clarity.

This correspondence advances the literature by emphasizing POFS in cardiac surgery, which is relatively neglected compared to pain and opioid use. By proposing continuous PIFB and broader patient inclusion, we address limitations in Wang et al.’s study and suggest practical improvements for clinical practice. The key learning points are: (1) early POFS is reduced with PIFB due to effective pain management and decreased inflammatory markers; (2) the benefits of continuous PIFB may last longer than 5 days; (3) the generalizability of the findings increases when diverse cohorts, including obese patients, are included; and (4) the inclusion of sleep and mood evaluation alongside other parameters.

In summary, Wang et al.’s focus on POFS is pioneering, but incorporating continuous PIFB, diverse cohorts, and functional cardiac assessments could enhance its role in improving cardiac surgery recovery protocols.

## Data Availability

This manuscript is a comment. Don’t need a data availability statement. However, all the data from the current study are publicly available.
